# Supplementary material for: Photocurable Thiol–yne Alginate Hydrogels for Regenerative Medicine Purposes
Source: Polymers (Basel). 2022 Nov 3;14(21):4709. doi: 10.3390/polym14214709 (PMC9654832; doi:10.3390/polym14214709)

Photocurable Thiol-Yne Alginate Hydrogels for Regenerative Medicine Purposes

Michael Zanon[1,2], Laura Montalvillo-Jiménez[2], Paula Bosch[2], Raquel Cue-López[2,3], Enrique Martínez-Campos[2,3], Marco Sangermano[1] and Annalisa Chiappone[4*]

1 Dipartimento di Scienza Applicata e Tecnologia, Politecnico di Torino, C.so Duca degli Abruzzi 24, 10129 Turin, Italy

2 Departamento de Química Macromolecular Aplicada, Instituto de Ciencia y Tecnología de Polímeros, Consejo Superior de Investigaciones Científicas (CSIC), C/Juan de la Cierva 3, Madrid, 28006, Spain

3 Grupo de Síntesis Orgánica y Bioevaluación, Instituto Pluridisciplinar (UCM), Unidad Asociada al ICTP, IQM (CSIC), Paseo de Juan XXIII 1, Madrid, 28040, Spain

4 Dipartimento di Scienze Chimiche e Geologiche, Università degli studi di Cagliari, Via Università 40, 09124 Cagliari, Italy

* Correspondence: annalisa.chiappone@unica.it

SUPPORTING INFORMATION

Figure S1 Integration of NMR spectra

A)

SA: ^{1}H NMR (300 MHz, D_2O) δ 5.04 (s, 1H, H-1-Guluronic acid), 4.28-3.54 (m, 8H, 8CH-SA).

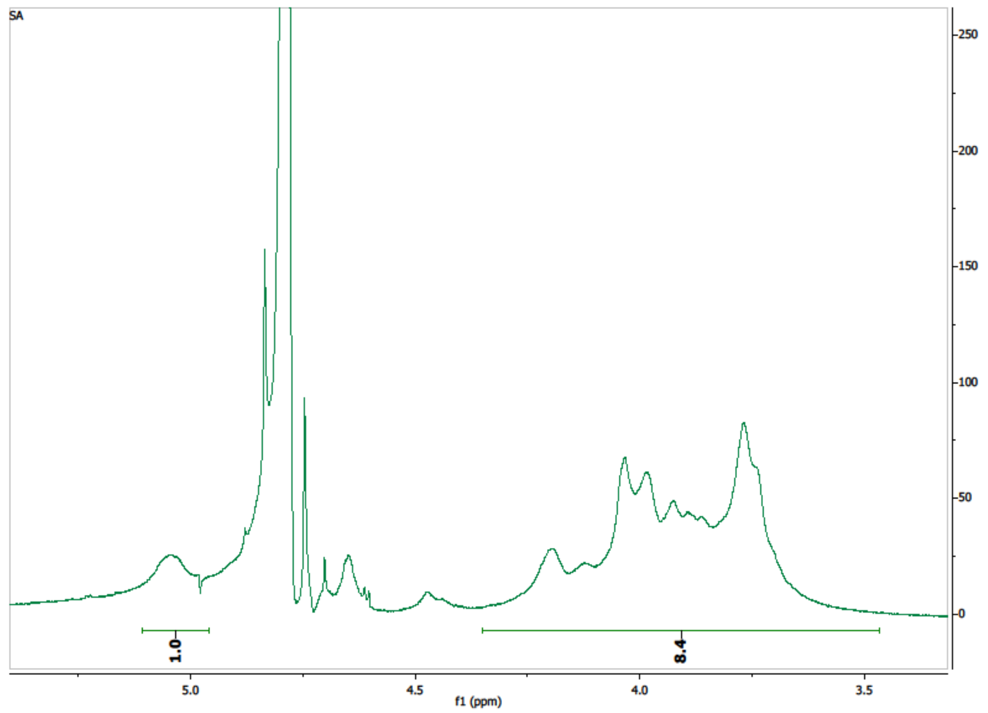

B)

SA-PA 1: ^{1}H-NMR (300 MHz, D_2O) δ 5.06 (br s, 1H, H-1-Guluronic acid), 4.37-3.62 (m, 11H, 8CH-SA, CH-PA), 3.44 (br s, 0.5 H, CH_2-PA).

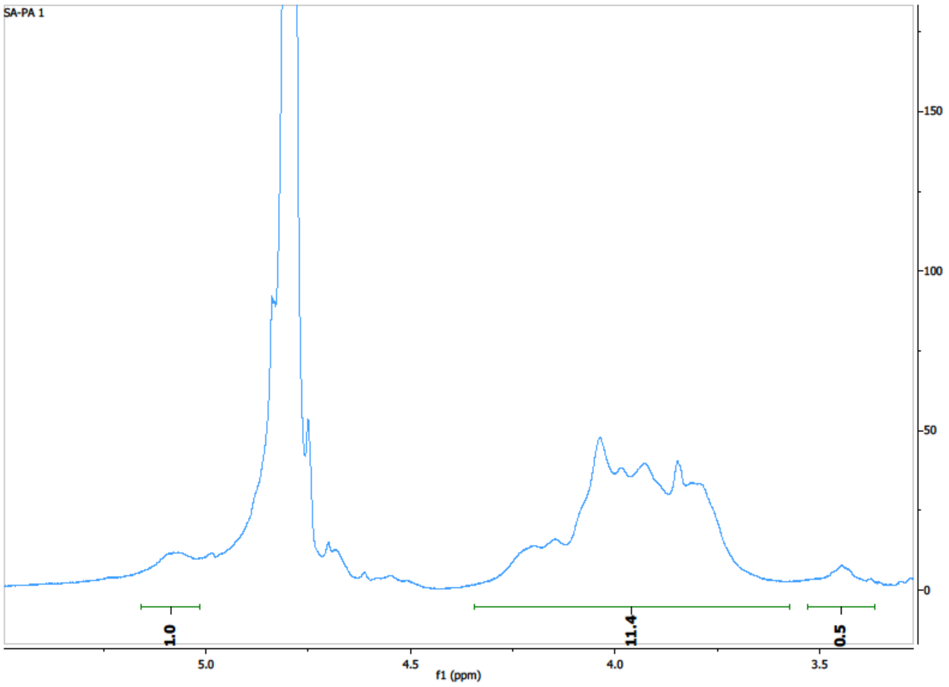

C)

SA-PA 4: NMR spectra were recorded at 318 K, using D2O as solvent, on an Agilent SYSTEM 500 NMR spectrometer (1H 500 MHz, 13C 125 MHz) equipped with a 5-mm HCN cold probe

^{1}H-NMR (500 MHz, D_2O) δ 5.05 (br s, 1H, H-1-Guluronic acid), 4.39-3.60 (m, 11H, 8CH-SA, CH-PA), 3.42 (br s, 2 H, CH_2-PA).

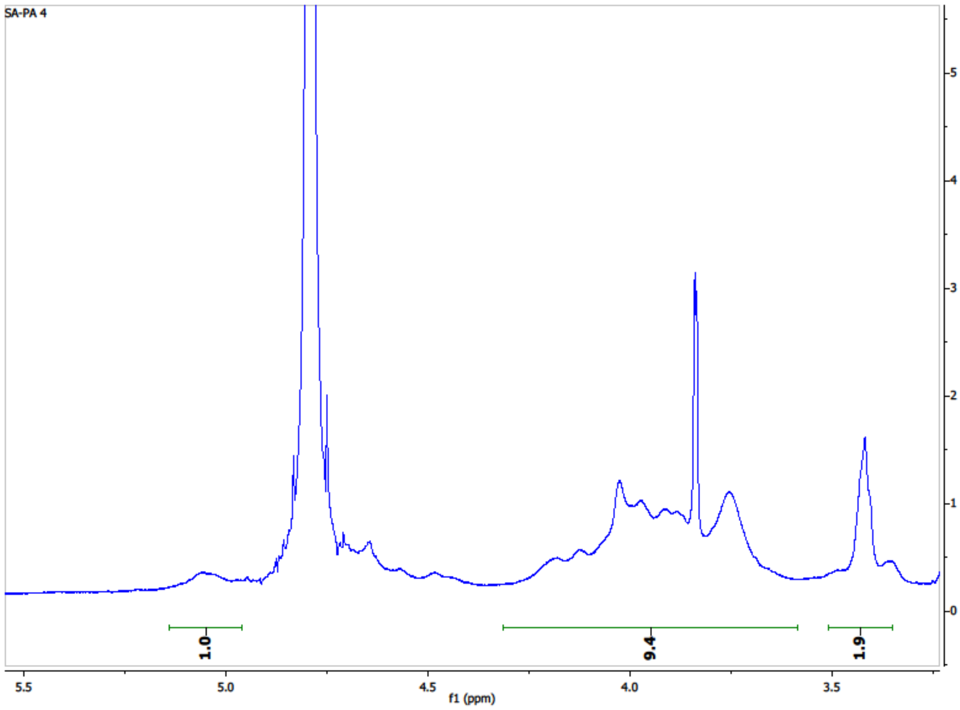

Supplement: Supplementary file 1 [file polymers-14-04709-s001.zip › polymers-1974466-supplementary.pdf]
